# Deep Learning Models to Predict Diagnostic and Billing Codes Following Visits to a Family Medicine Practice: Development and Validation Study

**DOI:** 10.2196/64279

**Published:** 2025-03-07

**Authors:** Akshay Rajaram, Michael Judd, David Barber

**Affiliations:** 1 Department of Family Medicine, Queen's University, 220 Bagot Street, Kingston, ON, K7L 3G2, Canada, 1 613 533 9300; 2 Department of Emergency Medicine, Queen's University, Kingston, ON, Canada

**Keywords:** machine learning, ML, artificial intelligence, algorithm, predictive model, predictive analytics, predictive system, family medicine, primary care, family doctor, family physician, income, billing code, electronic notes, electronic health record, electronic medical record, EMR, patient record, health record, personal health record

## Abstract

**Background:**

Despite significant time spent on billing, family physicians routinely make errors and miss billing opportunities. In other disciplines, machine learning models have predicted Current Procedural Terminology codes with high accuracy.

**Objective:**

Our objective was to derive machine learning models capable of predicting diagnostic and billing codes from notes recorded in the electronic medical record.

**Methods:**

We conducted a retrospective algorithm development and validation study involving an academic family medicine practice. Visits between July 1, 2015, and June 30, 2020, containing a physician-authored note and an invoice in the electronic medical record were eligible for inclusion. We trained 2 deep learning models and compared their predictions to codes submitted for reimbursement. We calculated accuracy, recall, precision, *F*_1_-score, and area under the receiver operating characteristic curve.

**Results:**

Of the 245,045 visits eligible for inclusion, 198,802 (81%) were included in model development. Accuracy was 99.8% and 99.5% for the diagnostic and billing code models, respectively. Recall was 49.4% and 70.3% for the diagnostic and billing code models, respectively. Precision was 55.3% and 76.7% for the diagnostic and billing code models, respectively. The area under the receiver operating characteristic curve was 0.983 for the diagnostic code model and 0.993 for the billing code model.

**Conclusions:**

We developed models capable of predicting diagnostic and billing codes from electronic notes following visits to a family medicine practice. The billing code model outperformed the diagnostic code model in terms of recall and precision, likely due to fewer codes being predicted. Work is underway to further enhance model performance and assess the generalizability of these models to other family medicine practices.

## Introduction

Previous research has revealed that family physicians spend nearly 50% of their day on electronic medical records (EMRs) and that most of this time is spent on administrative tasks, including documentation of notes and billing [1]. Physicians in the United States and Canada spend an average of 3.4 hours and 2.2 hours per week, respectively, writing, reviewing, submitting, and disputing claims with significant financial losses [2,3]. Tseng et al [4] estimated total professional billing costs for a typical primary care physician at nearly US $100,000 using time-driven activity-based costing. In addition to billing costs, attending and resident family physicians routinely make significant errors and miss opportunities in the context of billing [5,6].

While reasons for these errors and missed opportunities are multifactorial, experts have focused on a lack of education as a primary driver [7,8]. However, the literature demonstrates that even when robust practice management curricula are introduced, billing performance does not improve significantly [9]. Moreover, experienced attending family physicians report challenges with complex billing tasks, suggesting that accumulated experience does not enhance comfort [10].

Given limitations in education and training as quality improvement interventions, other system-focused strategies are warranted [11]. One potential solution is the use of artificial intelligence to predict diagnostic and billing codes from notes. Kim et al [12] demonstrated 87% accuracy of their machine learning model to predict Current Procedural Terminology (CPT) codes for spine surgery from operative dictations. Another study demonstrated 98% accuracy of a neural network in assigning CPT codes to pathology reports [13].

Little is known about whether similar approaches would work in family medicine, where presenting problems and assessments are highly diverse. Our primary objective was to assess the accuracy of machine learning models in predicting diagnostic and billing codes from the notes recorded in EMRs for visits to family physicians. Based on similar studies, we hypothesized that both the diagnostic and billing code models would generate predictions with at least 90% accuracy [12-14].

## Methods

### Design and Setting

We conducted a retrospective model development and validation study at a large academic Family Health Team (FHT) in Ontario, Canada, with approximately 50,000 visits per year. The FHT is in a more urban setting with a patient census of approximately 21,000 rostered to 26 attending physicians. Approximately 55-60 first-year resident physicians rotate through annually.

Faculty physicians at this site are primarily compensated through capitation payments but also submit invoices for individual visits as part of the province’s Family Health Organization funding model. A single-payer system predominates, with most invoices submitted to the provincial health insurance plan for reimbursement. A minority of invoices are submitted to other insurance plans, including the Workplace Safety and Insurance Board or a third party (eg, Blue Cross) or directly to patients. In addition to faculty and residents, locum physicians provide clinical coverage and submit invoices for individual visits.

Following a patient visit, physicians document their note in an EMR often in the SOAP (subjective, objective, assessment, plan) format. To submit an invoice, physicians must select 1 or more diagnostic codes and 1 or more billing codes. Invoices are compiled electronically in the EMR, reviewed by FHT billing personnel, and subsequently submitted to the provincial health insurance plan for payment every month.

Oscar is the EMR used in this study, and it contains a combination of structured and unstructured data organized into modules. Structured fields include demographics, billing (invoice number, diagnostic codes, billing codes, and billing history), preventative interventions, disease registry, laboratory results, measurements, consultations, allergies, medications, risk factors, and family history. Unstructured fields include social history, medical history, and free text chart notes.

### Ethical Considerations

This study received local research ethics board approval (FMED-6780‐20) from Queen’s University Health Sciences Research Ethics Board. The approval covered secondary analyses of these data without additional consent. Physicians were given an opportunity to censor specific patients or opt out of participation. Following the opt-out process, data of the included patients were exported as a flat file and stored on a secure server meeting local privacy requirements. Data were subsequently anonymized and deidentified during the preprocessing stage.

### Participants and Sampling

Between July 1, 2015, and June 30, 2020, 245,045 visits containing a documented note and an invoice submitted to the provincial health insurance plan for payment were eligible for inclusion. The included data comprised invoices containing diagnostic and billing codes and information about the status of reimbursement, corresponding visit information including the length of appointment, the date of birth of the patient, the patient’s gender, and the physician’s free text note for the visit. We excluded visits that had invoices that were not paid or were deleted.

### Data Preprocessing

We first transformed data into a Pandas Dataframe for additional preprocessing, including deidentification, linkage of appointments with relevant features, feature scaling, and clinical text processing.

#### Deidentification

Data were initially in an identifiable form but were anonymized using an automated PERL-based deidentification software package designed for free-text medical records [15]. The software uses a combination of lexical look-up tables, regular expressions, and simple heuristics to locate traditional personal health information, including common names and date variations [15]. This information was then tokenized and removed.

#### Linking of Appointments With Relevant Features

In Oscar, appointments are associated with both billing and diagnostic codes and contain the length of time for the visit. We linked appointments as an entity with the following data:

1. Demographic data for the patient, including age at the time of the appointment and gender.
2. Free text chart notes from the relevant table: Oscar does not relate a single note entity to an appointment. Notes were linked with their corresponding appointment by an exact match of dates. The signed and verified note by the attending physician was matched in cases of multiple notes from 1 session.
3. Historical diagnostic codes listed 6 months preceding the appointment date: these codes were recorded, and the frequency of the codes was summed.

#### Feature Scaling for Structured Data

To facilitate the use of neural networks with a gradient descent approach, we scaled our data to achieve values between 0 and 1. We used different feature scaling for different fields: (1) *MinMax scaler* from Scikit-learn for age and appointment duration [16]; (2) binary encoding for male and female; and (3) *MultiLabelBinarizer* for one-hot encoding of historical diagnostic codes [16].

#### Clinical Text Processing

We applied the following preprocessing steps to overcome common challenges encountered with clinical text, including domain-specific language, spelling mistakes, and redundant phrases [17]:

1. Stop words: we removed stop words (eg, “a,” “the,” “is”) from the text using the list contained in the NLTK package in Python [18].
2. Oscar-specific domain language: clinical notes signed by physicians include a phrase “SIGNED AND VERIFIED BY,” so *regex* was applied to remove this phrase from the text.
3. Deidentification tokens: the deidentification tool replaces all personally identifiable information with specific tokens. We removed these tokens from the text.
4. Spelling mistakes: we corrected potential spelling errors by applying the Symmetric Delete spelling correction algorithm (SymSpell) with the MEDLINE unigram dictionary, which includes over 28 million unique terms.
5. Punctuation: we removed punctuation from the text.
6. Vectorization: we vectorized the text into a sequence of numbers in the *term frequency–inverse document frequency* format [19].

### Model Training and Testing

**Figure 1.**
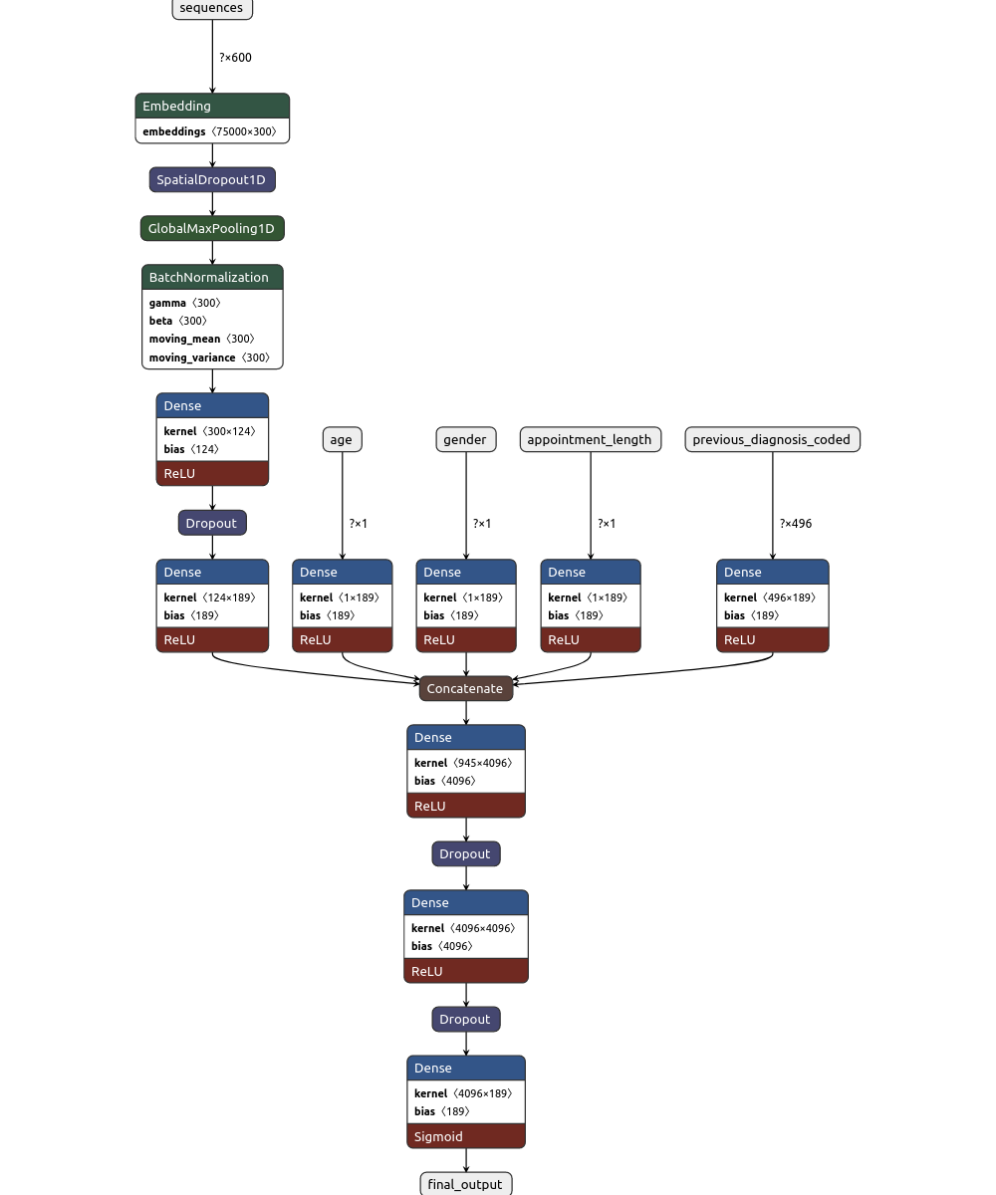
Graphical representation of model architecture. ReLU: rectified linear unit.

We used Tensorflow and Keras to construct one model each for the prediction of diagnostic codes and billing codes. Each model uses the same model architecture with the following layers. A graphical representation of the model architecture is presented in Figure 1.

One input layer for the vectorized note and 1 input layer are assigned for each structured data feature including age, gender, previous diagnostic codes, and appointment duration. For text classification, we used a submodel architecture called *fasttext* [20]. For structured data classification, we used a simple, fully connected, single-level Dense layer followed by a Dropout layer [21]. Weights were randomly set in the inputs. We then concatenated the text classification output layer and each structured data output layer and applied multiple layers of a Dense network followed by a Dropout layer with a rectified linear unit (ReLU) activation function. The final output layer contains a sigmoid activation function and returns multilabel outputs.

### Analysis

We divided data for model development into training, testing, and validation sets, using 70% (139,161/198,802) of notes for training and 30% (59,641/198,802) for testing and validation.

In the testing set, the diagnostic code model assigned 1 of 459 unique diagnostic codes while the billing code model assigned 1 of 157 unique billing codes. These codes are based on the Ontario Health Insurance Plan Schedule of Benefits for family medicine [22]. Each model initially returned a prediction score for each code ranging from 0 to 1. The prediction threshold to transform scores into labels (ie, the most likely diagnostic and billing code for the note) was selected by optimizing for the *F*_1_-score. The diagnostic and billing codes predicted by the deep learning models were compared to the codes selected by the clinician or updated by the FHT’s billing personnel that were ultimately billed to the health insurance plan.

Given the size of both datasets, we were unable to manually review and validate the diagnostic and billing codes of notes. However, the family medicine practice in our study benefits from having dedicated administrative staff who review invoices monthly and correct errors prior to submission for reimbursement.

Several metrics of model performance, including accuracy (correct predictions divided by total predictions), recall or sensitivity (true positives/[true positives+true negatives]), precision or positive predictive value (true positives/[true positives+false positives]), *F*_1_-score (2*true positives/[2*true positives+false positives+false negatives]), and area under the receiver operating characteristic curve, were calculated after testing using bootstrapping. We report 95% confidence intervals. Given the multiclass nature of diagnostic and billing code prediction and anticipated class imbalances, we report microaverages as a default unless otherwise specified. We generated performance metrics using *sklearn* in Python.

## Results

Of the 245,045 visits eligible for inclusion, 198,802 (81%) were included in model derivation, representing 32,425 unique patients. Three physicians opted out of participation in the study. Collectively, there were 448 unique note authors (faculty, physicians, resident physicians, or nurses). For training, 139,161 notes were used, while 29,820 and 29,821 notes were used for testing and validation, respectively. The mean length of notes was 195 (SD 102) words in the training, testing, and validation sets. The training, testing, and validation sets are compared in Table 1.

**Table 1.** Comparison of the training, testing, and validation datasets in model development.

		Training (n=139,161)		Testing (n=29,820)	Validation (n=29,821)
Ages, n (%)					
Patients aged 0-17 years		76,539 (55)		16,341 (54.8)	16,431 (55.1)
Patients aged 18-65 years		40,078 (28.8)		8707 (29.2)	8678 (29.1)
Patients aged >65 years		22,405 (16.1)		4771 (16)	4771 (16)
Sex, n (%)					
Male patients		85,027 (61.1)		18,160 (60.9)	18,370 (61.6)
Female patients		54,134 (38.9)		11,660 (39.1)	11,451 (38.4)
Notes, mean (SD)					
Note length (number of words)		194.7 (102.2)		195.0 (102.0)	194.7 (101.4)
Number of diagnostic codes per appointment		1.3 (0.6)		1.3 (0.6)	1.3 (0.6)
Number of billing codes per appointment		1.0 (0.1)		1.0 (0.1)	1.0 (0.1)
Codes, n (%)					
799		16,268 (11.7)		3426 (11.5)	3477 (11.7)
300		7779 (5.6)		1706 (5.7)	1706 (5.7)
916		6708 (4.8)		1440 (4.8)	1428 (4.8)
250		6666 (4.8)		1381 (4.6)	1425 (4.8)
401		5747 (4.1)		1223 (4.1)	1217 (4.1)
A007A		90,803 (65.2)		19,601 (65.7)	19,470 (65.3)
A001A		7139 (5.1)		1521 (5.1)	1563 (5.2)
G590A		6596 (4.7)		1378 (4.6)	1396 (4.7)
K005A		5887 (4.2)		1279 (4.3)	1235 (4.1)
G010A		4745 (3.4)		972 (3.3)	1041 (3.5)

**Figure 2.**
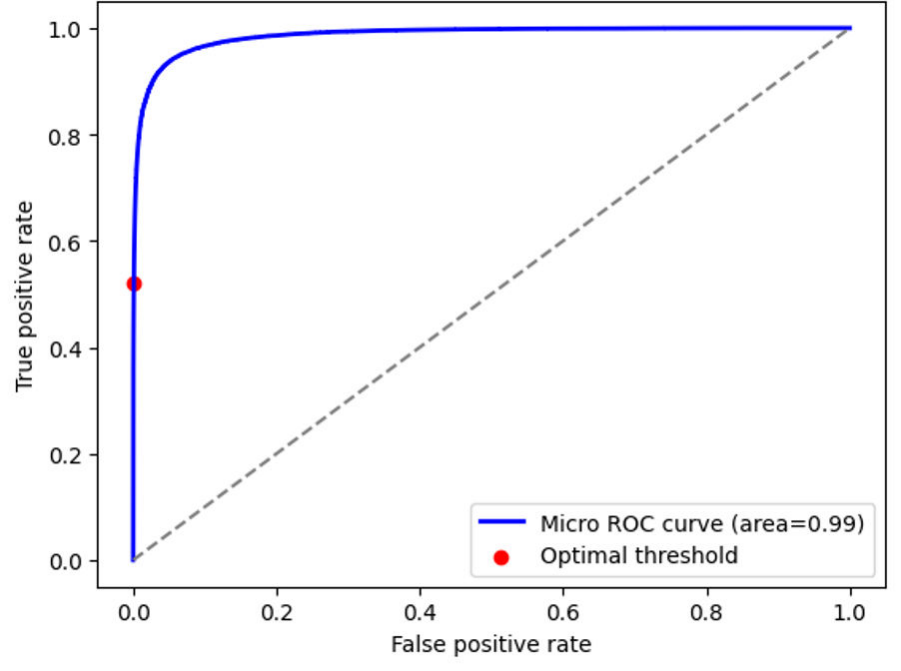
Area under the ROC curve for the diagnostic code model. ROC: receiver operating characteristic.

The overall accuracy of the diagnostic and billing code models were 99.8% (95% CI 99.79%‐99.80%) and 99.5% (95% CI 99.57%‐99.60%), respectively. The recall (sensitivity) was 49.4% (95% CI 49.07%‐51.77%) for the diagnostic code model and 70.3% (95% CI 68.68%‐72.17%) for the billing code model. The precision (positive predictive value) was 55.3% (95% CI 54.31%‐55.79%) for the diagnostic code model and 76.7% (95% CI 72.29%‐74.58%) for the billing code model. The *F*_1_-scores were 52.2% (95% CI 51.56%‐52.16%) and 73.4% (95% CI 72.29%‐74.58%) for the diagnostic and billing code models, respectively. Measures of model performance are reported in Table 2. The area under the receiver operating characteristic curves for the diagnostic and billing code models are shown in Figures2  3, respectively. The precision-recall curves are shown in Figures4  5, respectively.

**Table 2.** Measures of performance for the diagnostic and billing code models.

	Diagnostic code model (95% CI)	Billing code model (95% CI)
Accuracy, %	99.8 (99.79‐99.80)	99.5 (99.5‐99.60)
Recall, %	49.4 (49.07‐51.77)	70.3 (68.68‐72.17)
Precision, %	55.3 (54.31‐55.79)	76.7 (72.29‐74.58)
*F*_1_-score, %	52.2 (51.56‐52.16)	73.4 (72.29‐74.58)
AUC^a^	0.983 (0.9833‐0.9863)	0.993 (0.9921‐0.9943)

a AUC: area under the receiver operating characteristic curve.

**Figure 3.**
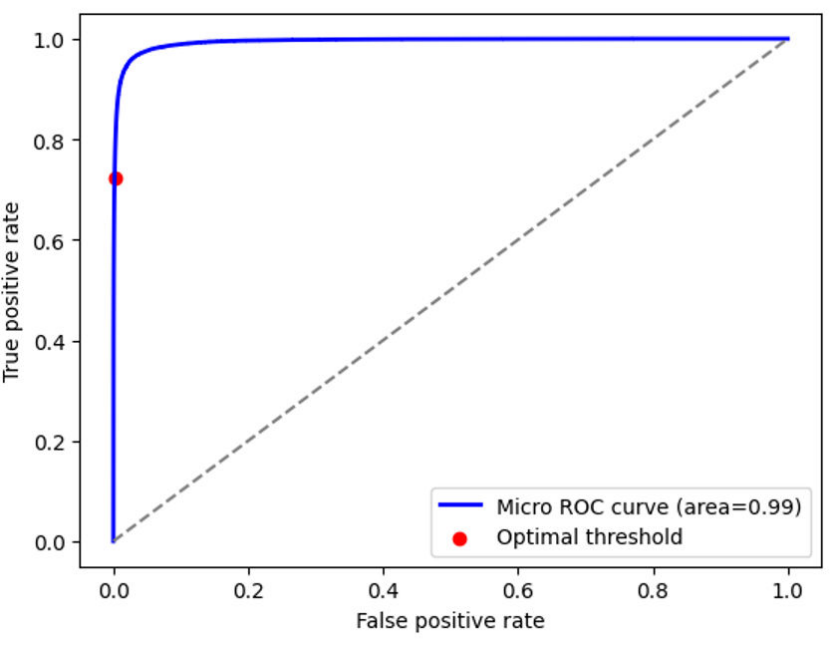
Area under the ROC curve for the billing code model. ROC: receiver operating characteristic.

**Figure 4.**
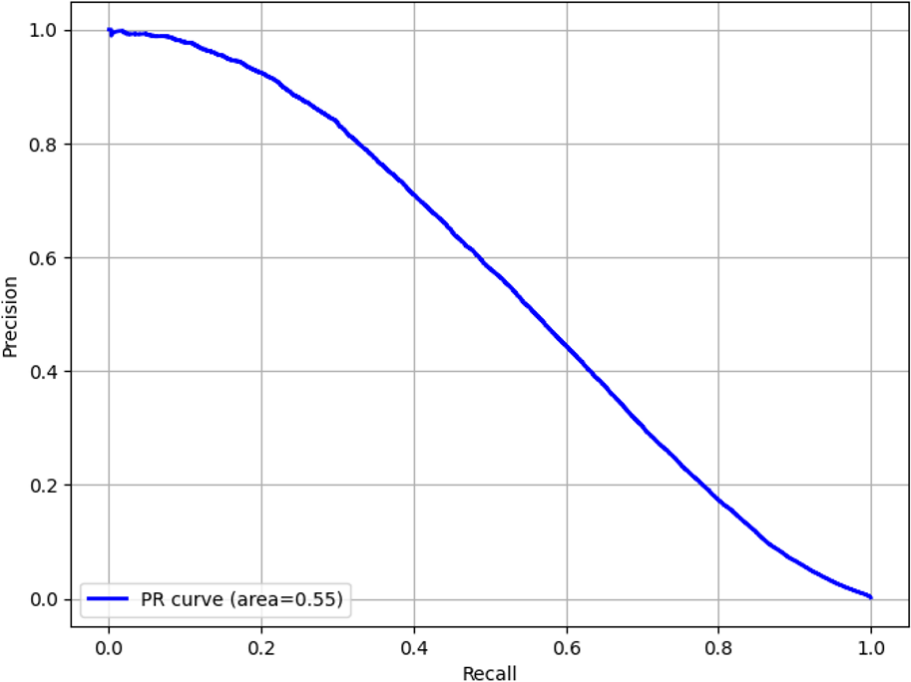
Precision-recall (PR) curve for the diagnostic code model.

**Figure 5.**
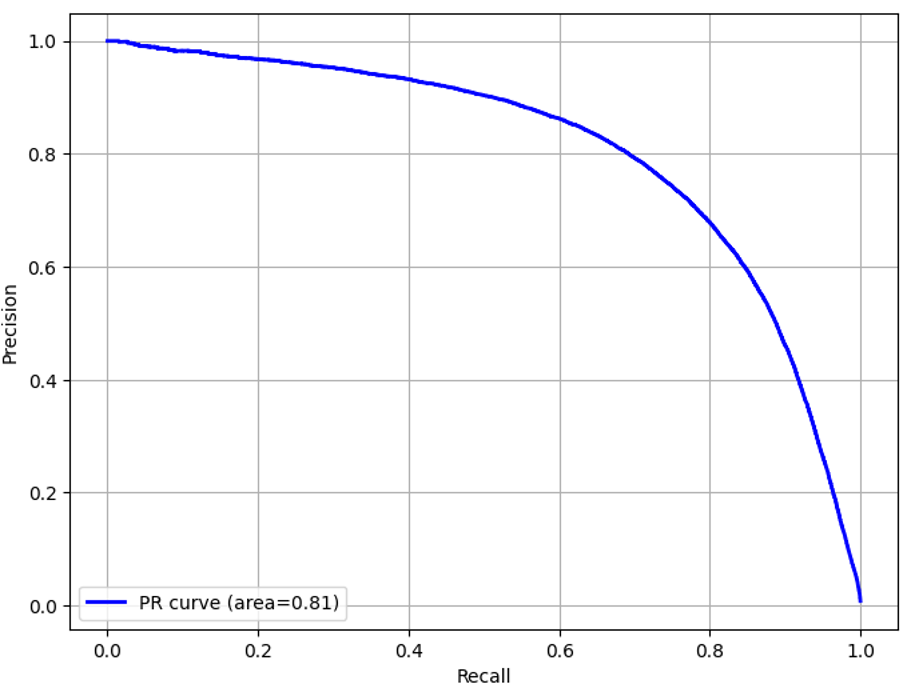
Precision-recall (PR) curve for the billing code model.

In the testing set, code 799 (“symptoms, signs and ill-defined conditions”) was the most commonly appearing diagnostic code (n=3425) followed by code 300 (“mental disorders – neuroses and personality disorders”; n=1707) and then code 916 (“well baby care”; n=1439). Code A007 (“intermediate assessment or well baby care”) was the most billed code (n=19,601). Code A001 (“minor assessment”) was the second most billed code (n=1520), followed by code G590A (“immunization – influenza agent”; n=1783). The top 10 most common diagnostic and billing codes and corresponding model performances are listed in Table 3.

**Table 3.** Prevalence and model prediction performance for the top 10 diagnostic and billing codes in the testing set.

	Description	Support, n	Precision, %	Recall, %	*F*_1_-score, %
Diagnostic code					
799	Symptoms, signs and ill-defined conditions	3425	78.3	63.5	70.1
300	Mental disorders – neuroses and personality disorders	1707	59.2	70.2	64.3
916	Well baby care	1439	83.9	92.2	87.8
250	Diabetes mellitus including complications	1382	73.7	82.8	78.0
401	Hypertension, essential	1222	62.4	68.2	65.2
650	Delivery – normal; pregnancy – uncomplicated; complications of pregnancy, childbirth and the puerperium – normal pregnancy	1206	86.2	92.8	89.4
847	Neck strain/sprain	856	51.1	57.5	54.1
311	Depressive or other non-psychotic disorder (not classified elsewhere)	790	53.6	53.4	53.5
844	Strains, sprains, and other trauma – knee, leg	685	51.4	65.7	57.7
787	Abdominal – pain, masses	639	45.4	47.0	46.2
Billing code					
A007A	Intermediate assessment or well baby care	19,601	85.7	89.6	87.6
A001A	Minor assessment	1520	45.1	46.5	45.8
G590A	Immunization – influenza agent	1378	91.1	63.9	75.1
K005A	Primary mental health care – individual care	1278	49.2	71.5	58.3
G010A	One or more parts of above without microscopy	972	58.5	63.2	60.8
K030A	Diabetic management assessment	920	66.8	84.4	74.6
P004A	Minor prenatal assessment	810	80.9	93.0	86.5
E430A	Pap (Papanicolaou) smear tray fee when performed outside of hospital	681	75.2	85.9	80.2
Q015A	Newborn care episodic fee	609	65.4	74.2	69.5
G365A	Pap (Papanicolaou) smear - periodic	583	69.9	90.1	78.7

## Discussion

### Principal Results

To our knowledge, this study is the first to report the development and internal validation of machine learning models for the prediction of diagnostic and billing codes in family medicine. While the models were highly accurate in terms of predictions, their recall and precision were much lower. These differences in performance are characteristic of multiclassification problems where high rates of overall accuracy are driven by higher classification of true negatives than identification of true positives. In the context of diagnostic and billing codes, however, correctly generating the relevant codes is much more useful than excluding irrelevant or inappropriate codes.

Unsurprisingly, the billing code model outperformed the diagnostic code model likely due to fewer codes being predicted. The lower precision and *F*_1_-score of the diagnostic code model suggest that the model struggles to correctly identify and classify true positive cases. There are a few possible explanations for this finding. First, the dataset was imbalanced with most diagnostic labels relating to ill-defined conditions (code 799), mental disorders (code 300), well baby care (code 916), and diabetes mellitus (code 250). Performance for these codes was noticeably better than for the overall dataset with recall ranging from 63%‐92% and precision ranging from 59%‐84%. Second, misclassification was also possible. Patients of the academic FHT where the study was conducted are known to be medically comorbid and socially complex. Consequently, encounter notes may yield several diagnostic labels; however, only 1 code may be selected for the visit.

Part of the challenge in selecting a diagnostic label for these encounters is observed among the top performing diagnostic codes. Although code 799 (“symptoms, signs and ill-defined conditions”) was the most frequent code in the dataset, recall was higher for several other codes, including codes 650 (“delivery – normal; pregnancy – uncomplicated; complications of pregnancy, childbirth and the puerperium – normal pregnancy”), 916 (“well baby care”) and 250 (“diabetes mellitus including complications”). These differences in performance are likely due to challenges in making sense of nonspecific symptoms in the case of code 799 as opposed to pregnancy (code 650) for a patient seeking antenatal care or a patient following up for diabetes (code 250).

We anticipated that the billing code model would perform better at predicting codes that were more frequently selected. The highest recall was for P004A, the billing code for minor prenatal assessment. Patients are seen several times during their pregnancy leading to the accumulation of these codes in historical invoices. Along with straightforward visit documentation, we suspect the model was able to predict the P004A code more fluently.

### Limitations

While our study is the first to derive and validate models to predict diagnostic and billing codes in family medicine, our results should be interpreted with caution. Our data were drawn from 1 academic FHT located in a single province and our models have not yet been externally validated. As a result, our findings may not be generalizable to other family medicine settings (eg, community or nonacademic) or other jurisdictions.

We observed heterogeneity in the performance of the model in classifying diagnostic and billing codes. Due to the size of the dataset, limited resources, and administrative constraints, we were unable to perform more detailed analyses relating to the interpretability and explainability for the diagnostic and billing code predictions. Such analyses may have uncovered factors influencing the model’s performance for each code and remain an important target for future work.

One factor that likely influenced performance is clinical note quality [23]. Generally, longer notes provide more information with the corollary being that more information tends to yield better predictions. However, longer notes may also contain more copied information, which may negatively impact natural language processing performance [23]. Similarly, previous work has shown differences in the documentation practices of trainee and attending physicians [24]. The notes of trainee physicians tend to be longer and more complete while attending physicians are most interested in the assessment and plan section of notes [24-26]. Critically, quality of documentation is challenging to assess, especially in family medicine settings where no validated tools exist.

### Comparison With Prior Work

Our findings are generally consistent with the results of previous studies. Using the open-source Medical Information Mart for Intensive Care III (MIMIC-III) database, various groups have developed machine learning models for the prediction of diagnostic (*International Classification of Diseases, Ninth Revision* [*ICD-9*]) codes from discharge summaries achieving micro *F*_1_-scores between 57.5‐58.9 [27]. Performance discrepancies between our diagnostic code model and the models in these studies may be attributed to differences between encounter notes and discharge summaries. The latter tend to be more comprehensive in capturing details regarding a patient’s initial presentation, their course and management in the hospital, and follow-up plans after discharge. These sections provide ample substrate on which to base predictions.

In the context of billing, Ye [13] developed a 3-layer neural network to predict CPT codes based on the diagnosis header and diagnosis recorded in pathology reports and achieved accuracy of 97.5%. However, their model only predicted 5 codes using text with a median length of 12 words. In contrast, Burns et al [14] developed a neural network to predict 232 CPT codes from procedural text with a mean word count of 10 words per text and achieved 82.1% accuracy. On average, notes in our study were approximately 10 times larger than those in the study by Burns et al, with a comparable number of billing codes and much higher accuracy [14].

### Implications

Despite the challenges associated with billing, including missed revenue opportunities and errors, the performance of our models suggest that more work is needed before machine-learned solutions for diagnostic and billing code prediction can be deployed in practice. Such work includes external validation with other academic and community family medicine clinics, prospective validation to compare performance with physicians, and the testing of generative pretrained transformer architectures.

Once completed, there are different ways these models could be embedded within existing billing workflows. Models could be integrated with existing EMRs providing diagnostic and billing code predictions to end-users in real-time. Physicians could review predictions before finalizing codes for submission. Alternatively, physicians could bill visits as they currently do with the model surfacing its predictions for encounters for which a code was missed or an error was made. Additionally, the model could be combined with rule-based approaches to reduce common errors.

### Conclusions

Our study is the first to describe the development and validation of machine learning models for the prediction of diagnostic and billing codes in family medicine. Model performance was heterogeneous and requires further analysis to uncover the factors associated with the prediction of specific diagnostic and billing codes. In addition to addressing model explainability, future work will incorporate additional structured data, consider the impacts of note characteristics and authorship on model performance, and explore validation in other family medicine settings.

## Abbreviations

CPT: Current Procedural Terminology
EMR: electronic medical record
FHT: Family Health Team
*ICD-9*: *International Classification of Diseases, Ninth Revision*
MIMIC-III: Medical Information Mart for Intensive Care III
ReLU: rectified linear unit
SOAP: subjective, objective, assessment, plan
